# Supplementary material for: Genomic variants from RNA-seq for goats resistant or susceptible to gastrointestinal nematode infection
Source: PLoS One. 2021 Mar 15;16(3):e0248405. doi: 10.1371/journal.pone.0248405 (PMC7959398; doi:10.1371/journal.pone.0248405)
Supplement: Attachment — Submitted filename: Comments.docx [file pone.0248405.s001.docx]

Article entitled, “Genomic variants from RNA-seq for goats resistant or susceptible to GI nematode infection is not acceptable in the present format and demands thorough revision. **Although the content of the article is good enough to publish in PLOS ONE but the MS has been written in a very casual way.** I request the authors to take up the challenge to revise the MS thoroughly for improvement. The following points should be taken into consideration:

1. Assignment of resistant and susceptible animals is confusing. The authors are requested to furnish details on this point.
2. In Discussion the meaning of first sentence is not clear. It appears that, they have worked with resistant/susceptible nematodes.
3. In my opinion total reorientation of Discussion is warranted.
(a) First paragraph should highlight the novelty of work stating research gap. Subsequently the authors should speak about the importance of the study and its uniqueness compared to other techniques for identification of resistant and susceptible animals.
(b) Second paragraph should emphasize “how SNPs have been exploited in caprine for climate adaptations study and other economic trait. Subsequently the authors are requested to mention other genetic studies to identify GIN resistant breeds. To mention a few are: Llie *et al*., 2018 (study in Romania and Hungary), Alam *et al*., 2019 published in J Anim Sci Biotechnol and Sharma  *et al*., 2012 published in International J. Anim. Vet. Sci. Apart from these if the authors find few more publications are requested to add in the Discussion.
(c) Third paragraph should emphasize the role of T cells for conferring adaptive immunity against GIN.
(d) Existing 3^rd^ paragraph sounds good and I feel this should be retained. However, I will be curious to see an elaboration of MAPK signaling pathway with a brief note on the observation in Yichang white goats.

Overall Assessment: Worthy to publish in **PLOS ONE** if the corrections are incorporated.
