# Supplementary material for: Genomic variants from RNA-seq for goats resistant or susceptible to gastrointestinal nematode infection
Source: PLoS One. 2021 Mar 15;16(3):e0248405. doi: 10.1371/journal.pone.0248405 (PMC7959398; doi:10.1371/journal.pone.0248405)
Supplement: Attachment — Submitted filename: revision note.docx [file pone.0248405.s002.docx]

**Revision Notes**

**Manuscript # PONE-D-20-03648**

Dear Editor,

First, we would like to thank you for giving us the chance for revision and to respond to the reviewers' comments. We have modified the manuscript according to the reviewers' recommendations and answered their questions in detail.

**Reviewer #1**

1. **Reviewer**/ This is a clear and interesting manuscript by a highly regarded group. However, some aspects of the methodology are not clear.

***R/ we made some changes to the methodology to make it more clear according to the raised points from the reviewer 1 and reviewer 2 as mentioned in details subsequently.***

2. **Reviewer**/ On line 71 the size of the cohort should be given.

***R/ the size of the cohort have been added (n=89) # line 74***

3. **Reviewer**/ Line 121 Groups of six what? Are they replicates from the same animal or were animals pooled?

***R/ The sentence has been corrected. In the revised version # lines 126-127 “groups of 6 technical replicates”***

4. **Reviewer**/ Lines 131-132 The justification for a frequency of over 50% is not clear.

***R/ we added justification. In the revised version # lines 137-138. “****to ensure the constant of each variant between individuals of the same group (group specific variant)****”***

**Reviewer #2**

1. **Reviewer**/ Article entitled, “Genomic variants from RNA-seq for goats resistant or susceptible to GI nematode infection is not acceptable in the present format and demands thorough revision. **Although the content of the article is good enough to publish in PLOS ONE but the MS has been written in a very casual way.** I request the authors to take up the challenge to revise the MS thoroughly for improvement. The following points should be taken into consideration.

***R/ we improved the revised manuscript taking into consideration the raised points from the reviewers.***

2. **Reviewer**/ Assignment of resistant and susceptible animals is confusing. The authors are requested to furnish details on this point.

***R/ we added details on this point. # lines 67-74 and lines 155- 159***

3. **Reviewer**/ In Discussion the meaning of first sentence is not clear. It appears that, they have worked with resistant/susceptible nematodes.

***R/ we modified the sentence. # lines 273- 275.***

4. **Reviewer**/ In my opinion total reorientation of Discussion is warranted.
(a) First paragraph should highlight the novelty of work stating research gap. Subsequently the authors should speak about the importance of the study and its uniqueness compared to other techniques for identification of resistant and susceptible animals.

***R/ The raised point have been taken into account # lines 240 -255***

(b) Second paragraph should emphasize “how SNPs have been exploited in caprine for climate adaptations study and other economic trait. Subsequently the authors are requested to mention other genetic studies to identify GIN resistant breeds. To mention a few are: Llie *et al*., 2018 (study in Romania and Hungary), Alam *et al*., 2019 published in J Anim Sci Biotechnol and Sharma  *et al*., 2012 published in International J. Anim. Vet. Sci. Apart from these if the authors find few more publications are requested to add in the Discussion.

***R/ second paragraph have been rebuilt taking into account the raised point. # lines 256 -277***

(c) Third paragraph should emphasize the role of T cells for conferring adaptive immunity against GIN.

***R/ The raised point have been taken into account # lines 288 -302***

(d) Existing 3^rd^ paragraph sounds good and I feel this should be retained. However, I will be curious to see an elaboration of MAPK signaling pathway with a brief note on the observation in Yichang white goats.

***R/ The raised point have been taken into account # lines 303 -310***

**Journal Requirements:**

Raw sequence reads uploaded to a stable, public repository (NCBI BIOPROJECT)

<http://www.ncbi.nlm.nih.gov/bioproject/667825>

Accession: PRJNA667825

ID: 667825
